# Evaluation of the COVID-19 Lockdown-Adapted Online Methodology for the Cytology and Histology Course as Part of the Degree in Veterinary Medicine

**DOI:** 10.3390/vetsci9020051

**Published:** 2022-01-27

**Authors:** Ana Balseiro, Claudia Pérez-Martínez, Paulino de Paz, María José García Iglesias

**Affiliations:** 1 Departamento de Sanidad Animal, Facultad de Veterinaria, Universidad de León, 24071 León, Spain; abalm@unileon.es (A.B.); mjgari@unileon.es (M.J.G.I.); 2 Departamento de Biología Molecular, Facultad de Veterinaria, Universidad de León, 24071 León, Spain; ppazc@unileon.es

**Keywords:** cytology and histology teaching–learning, presential evaluation (face-to-face), online evaluation, COVID-19, virtual microscopy, veterinary medicine

## Abstract

The COVID-19 pandemic and lockdown brought numerous teaching challenges requiring innovative approaches to teaching and learning, including novel modes of content delivery, virtual classrooms, and online assessment schemes. The aim of this study is to describe and assess the efficacy of the methods implemented at the University of León (Spain) to adapt to lockdowns in the context of the Cytology and Histology (CH) course for veterinary medicine undergraduate students. To evaluate the success of lockdown-adapted methodologies, we used inferential statistical analysis to compare the academic outcomes of two cohorts: 2018–2019 (traditional face-to-face—presential—learning and evaluation) and 2019–2020 (some face-to-face and some online lockdown-adapted learning and online lockdown-adapted evaluation). This analysis considered scores in both theoretical and practical exams and students’ final subject score. We also evaluated the number of logs onto the Moodle platform throughout the 2019–2020 period, as well as performing a student satisfaction survey in both courses. The use of explanatory pre-recorded lectures, continuous online self-assessment tests, and virtual microscopy (VM) may have produced significant improvements in the acquisition of histology competencies among students in the lockdown cohort. However, we need to implement further strategies to improve the assessment of students’ true level of knowledge acquisition. According to the student feedback, VM is a well-accepted resource that is perceived as a flexible and enjoyable tool to use. However, while students found that the resource enhances their ability to learn about microscopic structures, they felt that it should not completely replace optical microscopy.

## 1. Introduction

Competency-based veterinary education is an academic innovation implemented in Europe to ensure that graduates meet their professional and social needs [1]. The focus on student-centered learning and teaching outcomes offers a series of benefits to students, institutions, and society. However, the establishment of a shared, comprehensive, competency-based framework in veterinary education is under constant review. Currently, areas of innovation (research into teaching and learning) in veterinary education focus on assessment tool validation, developing new learning resources to support different learning styles, and evaluating the effectiveness of technology in the acquisition of the required professional competencies.

Recent years have witnessed an explosive growth in the use of educational technology in veterinary education in order to foster an active learning environment. The practical element of cytology and histology (CH), which aims to teach learners to identify different cells, tissues, and organs, has traditionally been undertaken using glass slides and an optical microscope (OM), with the use of web-based resources being limited to static images. Students often complained that they could only examine the histological slides during practical sessions, which, in their opinion, did not given them enough time to acquire sufficient histological interpretation skills. To address this, and to improve the teaching of histology and pathology, over the last five years, several veterinary faculties have begun integrating virtual microscopy (VM) into their study programs [2,3,4]. The teaching challenges brought about by the COVID-19 crisis, particularly the necessity of an abrupt switch to remote teaching [5], have highlighted the important role of VM in imparting practical skills in medical histology and pathology education [6,7,8].

Our research aims to assess the quality of histology teaching and the lockdown-adapted student evaluation process that was developed during the COVID-19 pandemic, with a particular focus on VM as a key tool for practical teaching. Our original approach is based on comparing the methodologies used in the 2018–2019 course (traditional face-to-face—presential—learning and evaluation) and 2019–2020 course (some face-to-face and some online lockdown-adapted learning and online lockdown-adapted evaluation). To achieve our aim, we analyzed student outcomes with respect to the acquisition of the required competencies in CH by Veterinary Medicine undergraduate students at the University of León (León, Spain). The specific aims of the present study were (i) to describe the lockdown-adapted teaching and learning methodology developed in response to the COVID-19 pandemic that affected the 2019–2020 student cohort, (ii) to assess their efficacy by means of a comparison of student outcomes between the 2019–2020 cohort and the 2018–2019 cohort that received the traditional face-to-face course, (iii) to evaluate the potential weaknesses of the online methodology (teaching and evaluation), and (iv) to assess the students’ level of satisfaction after using VM as either a complementary or main tool for practical training, during, respectively, the traditional face-to-face course and the lock-down-adapted course.

## 2. Materials and Methods

### 2.1. Overview of Cytology and Histology at the University of León

CH is a mandatory subject that is taught in the second semester (from February to July) of the first year of the Degree of Veterinary Medicine at the University of León (León, Spain). The course covers the morphological study of healthy cells (cytology), and the association among cells to form tissues (histology), as well as organs and systems (microscopic anatomy). CH carries a total of six European credits (ECTS), for which the final score is calculated as follows: 27% theory, 55% practical knowledges, 10% supervised work, 4% online tests to be scored and 4% attendance.

Pre-COVID, the students would complete 45 h of laboratory sessions, where they gain practical training in histological techniques (1 h), histology (16 h), and microscopic anatomy (28 h) (Table 1).

Each laboratory session on Histology and Microscopic Anatomy (2 h/session) consisted of an explanation by the lecturer and subsequent visualization by the students (approximately 45 min) of a set of the corresponding histological slides using a single-headed OM. Any questions brought up by students would also be answered by the lecturer in the practical part of the session. In the 2018–2019 academic year, VM was introduced as a complementary tool to improve teaching and provide students with continuous access to microscopy samples for study. Students could contact their lecturers via email or meet face-to-face throughout the course.

In Spain, the COVID-19 lockdown started on 14 March 2020. At this point, the cytology and histology sections of the course had already been taught, meaning that the main impact was seen during the Microscopic Anatomy section of the course. Teaching was immediately adapted to the new situation with the provision of online resources including pre-recorded lectures and VM on Moodle (Table 1). The Moodle platform is a free and open-source learning platform that educational institutions use to deliver courses and learning material to students. Moodle stands for “Modular Object-Oriented Dynamic Learning Environment”, and is distributed under the GNU General Public License. The platform has different tools, including: generating data such as online logs (i.e., views and messages), the participation of students in different activities and conducting surveys. The pre-recorded lectures followed a similar pattern to face-to-face classroom teaching, containing an explanation of theoretical concepts linking to applied histology using relevant images of organ tissues. VM became the key tool for practical training, totally replacing the use of conventional microscopy in the laboratory. Students’ problems were solved via email.

### 2.2. Preparation of Virtual Microscopy Slides

Histological slides from different tissues and organs were scanned at 400× magnification using the whole-slide scanning facility on an Olympus BX51^®^ (Olympus, Osaka, Japan) light microscopy. This system created high-resolution digital images of tissue sections, and these “virtual slides” could then be distributed to students through the Unileon network using hyperlinks available through the Moodle platform. Students could use Adobe Flash Player to view the virtual slides, which could be studied with 1.5× to 40× magnification.

An explanatory video was made available on the Moodle platform to train students in the use of VM for the examination of histological slides. Students could access the virtual slides any day and at any time from any computer connected to the Internet. Any problems that were encountered were solved by communication with lecturers via email.

### 2.3. Study Design and Participants

This quasi-experimental study was carried out using a corpus of students completing the CH course as part of their degree in Veterinary Medicine. The outcomes for two cohorts were compared: those who studied the course in 2018–2019 and received traditional face-to-face teaching with presential evaluation (*n* = 113) and those who studied the course in 2019–2020 and initially experienced face-to-face teaching followed by a period of online teaching and fully online evaluation (*n* = 114).

Lockdown-adapted teaching and student evaluation methods were compared with those used in traditional (face-to-face) course (Table 1 and Table 2). Both traditional and lockdown-adapted courses were taught by the same lecturers.

Our two student cohorts were non-random samples of convenience; thus, their homogeneity is not guaranteed. In this way, the results reported here must be interpreted strictly within the limits of this study.

### 2.4. Methods for Evaluating Students’ Acquisition of Required Competencies

Traditional student evaluation methods in CH included two written theory exams, both comprising short-answer questions, one assessing understanding of tissue components and the other assessing understanding of organ components (Table 2). Practical knowledge was assessed by two further written exams: (1) one in the classroom concerning the identification of cells, tissues (Table 2, image a), and organs (Table 2, image b) using projected static microscopic images, and (2) a second one in the laboratory concerning the identification and description of organs by examining histological slides under an OM (Table 2, image c).

During the COVID-19 lockdown, assessment followed the same four exam formats as before; however, rather than taking place in-person, exams were completed online on Moodle (Table 2, images d–f). The online assessment procedure was designed to prevent communication between students and minimize opportunities to consult external sources of assistance. Measures included: (a) dividing students into random groups that accessed the exam within a 1-min interval of each other, with the students being told to which group they belonged immediately before the exam started; (b) giving each group a different exam; (c) establishing the optimal time limit for completing each exam as the time taken for the lecturer to complete it multiplied by 1.5; (d) randomizing question order on every exam so that no groups had exactly the same exam; (e) designing the exam so that there were two pages, with half the questions on each, and when students moved from one page to the next they were unable to go back to the previous page; and (f) giving penalty marks for incorrect answers.

In addition, both pre-pandemic and lockdown cohorts took four online self-assessment tests: one concerning cells, one about tissues, and two on organs. Correct answers were provided as feedback.

**Figure 1:**
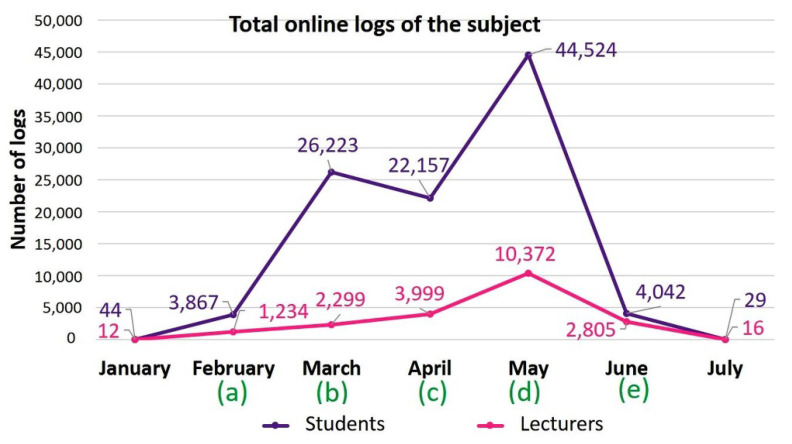
Total student and lecturer Moodle logins for the cytology and histology subject courses recorded throughout the lockdown-adapted course. Schedule of main activities: (a) Start date of cytology and histology teaching (10 February); (b) Start date of COVID-19 lockdown, which coincided with the start of microscopic anatomy teaching (14 March and 17 March, respectively); (c) Date of first term exam (28 April); (d) Date of second term and final exams (25 May); (e) Exams for students who failed the subject (18 June).

In the pre-pandemic course, attendance represented 4% of students’ final score and, during lockdown, this component was replaced by giving students the opportunity to complete a number of voluntary, self-assessed, non-scored online tests. Tests consisted of 4–5 questions on the topics covered by the pre-recorded lectures that were made available during lockdown. The tests were available for a limited time (5 days), and correct answers were provided as feedback once the questionnaire was closed. Students were able to email the lecturer responsible for each topic to resolve any problems. Figure 1 shows the Moodle activity recorded for the CH course in both the time leading up to and after the COVID-19 lockdown. The schedule of main activities is also indicated, and both student and lecturer activity is given in terms of the total number of logs onto the Moodle platform throughout the period of interest.

### 2.5. Criteria for Evaluating Students’ Acquired Knowledge

The following parameters were used to identify differences in learning outcomes between the traditional and the lockdown-adapted courses:

Monitoring scores obtained in the theoretical exams (presential exams versus online Moodle exams). Students could score from 0 to 12 points out of a total of 100 points in the cytology and histology examination, and from 0 to 15 points out of a total of 100 points in microscopic anatomy.

Monitoring scores obtained in the practical exams involving static images (presential exams versus online Moodle exams). Students could score from 0 to 15 points out of 100 in cytology and histology, and from 0 to 25 points out of 100 in microscopic anatomy.

Monitoring scores obtained in the practical exams involving microscope use to identify and describe organs (presential exams using OM versus online exams using VM). Here, students could score from 0 to 15 points out of a total of 100 points.

Final scores for the whole course adjusted to a 10-point scale, where 0 corresponded to the lowest score obtained in a given cohort. A further scaled score was obtained by grouping the 10-point scale into 4 categories, as follows: <5 for “fail”; 5 to 6.9 for “pass”; 7 to 8.9 for “good”; and ≥9 for “merit” (Real Decreto 1125/2003) [9].

### 2.6. Student Satisfaction Survey

Student perceptions of the use of VM in both the traditional pre-COVID course and the lockdown-adapted course were surveyed using a specially designed questionnaire on the Moodle platform. The questionnaire was voluntary and remained open for two months (June–July) after teaching was completed. The questionnaire comprised several items, evaluated on either a four-level scale (“strongly agree”, “agree”, “neutral”, and “disagree”) or a two-level scale (“yes” and “no”) and also included a “free comment” section (see Table 3 in the Results section).

### 2.7. Statistical Study

Inferential statistical analysis was used to compare student scores obtained from the traditional (2018–2019) and lockdown-adapted (2019–2020) evaluation procedures. The Kolmogorov–Smirnov test was used to assess the normality of quantitative variables and, because data were found to not follow a normal distribution, comparisons between cohorts were completed using the non-parametric Mann–Whitney U test. Results were expressed in terms of the median and interquartile range. To compare the percentage pass rates and qualitative score rates of students on each course, a Chi-square test or Fisher’s exact test were used, as appropriate. Statistical significance was established for *p* ≤ 0.05 (two-tailed), and differences where *p* < 0.10 were described as tendencies. Statistical analysis was conducted using SPSS software version 26 (SPSS Inc., Armonk, NY, USA: IBM Corp) for Windows. All data for this study were anonymized and treated confidentially according to the EU Data Protection Directive 95/46/EC. Before the study could be completed, a positive evaluation by the Ethics Committee of the University of León was obtained (reference number ETICA-ULE-031-2019).

## 3. Results

### 3.1. Characterization of Moodle Activity during the Lockdown-Adapted Course

**Figure 2:**
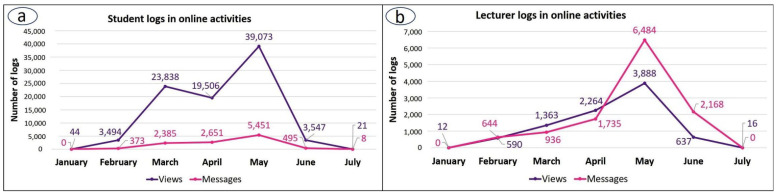
Data collected from Moodle for the cytology and histology elements of the lockdown-adapted course (2019–2020). Logs of views and messages by (**a**) students, and (**b**) lecturers.

Student access logs comprised the majority of Moodle logs for the whole period of the 2019–2020 course: 82.9% compared to 17.1% by lecturers, and a peak in Moodle access was recorded for both students and lecturers in May, when exams took place (Figure 1). In contrast to lecturer access logs, however, records of student logs also showed an earlier activity peak in March at the beginning of lockdown (Figure 2).

Additionally, throughout the 2019–2020 course, student activity was always greater for views of online activities than for messages. Lecturers, in contrast, had similar activity levels for both views and messages at all times, except during the exam months, where there was an increase in messages compared to views (May–June). This suggests that lecturers were responding to student questions about how the online exams were to be conducted (Figure 2).

**Figure 3:**
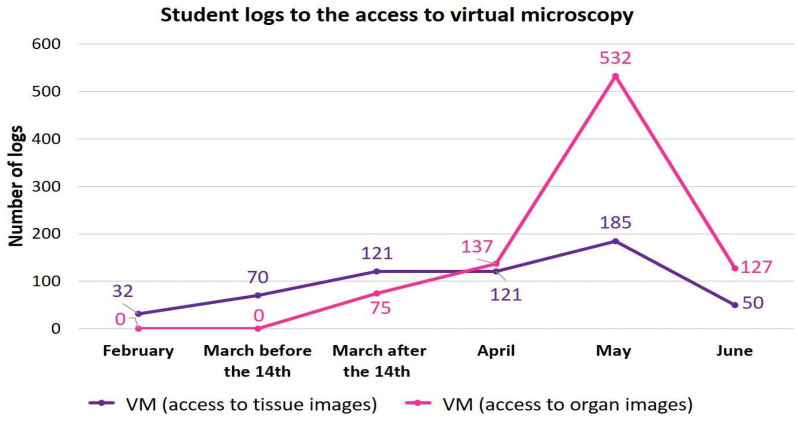
Data collected from Moodle throughout the lockdown-adapted course (2019–2020). Logs of students’ access to virtual microscopy (VM).

Referring to Figure 3, the start of microscopic anatomy teaching and lockdown occurred within days of each other and, as might be expected, records show increased levels of access by students to digital images of organs. A further increase in the use of these images occurred in May, during the exam period (Figure 3).

### 3.2. Student Outcomes: Comparing Face-to-Face and Lockdown-Adapted Evaluation Procedures

**Figure 4:**
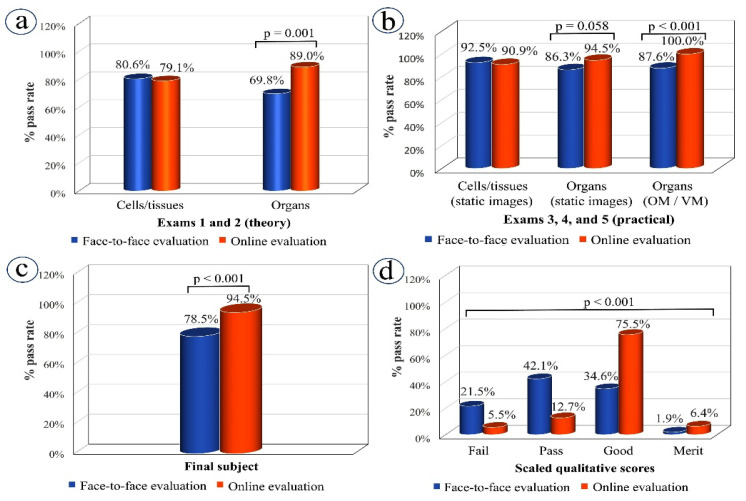
Students’ outcomes: a comparison between the 2018–2019 cohort examined in-person and the 2019–2020 lockdown cohort examined online. (**a**) Percentages of students passing the two theoretical exams (exams 1 and 2); (**b**) Percentage of students passing the three practical exams (exams 3, 4, and 5); (**c**) Percentage of overall passes in the cytology and histology course; (**d**) Percentages of final scaled qualitative scores for both student cohorts. Fisher’s exact test (**a**–**c**) and Chi-square test (**d**) were applied. OM, optical microscopy; VM, virtual microscopy.

Looking first at performance in the theory exams, both student cohorts (2018–2019 and 2019–2020) received traditional, face-to-face teaching for the cytology and histology course component and statistical analysis revealed that the percentage of students who passed exam 1 (cells and tissues) was similar when they were examined in-person and online (exams 1a and 1b, respectively). In contrast, the pass rate for exam 2 (on organs) was significantly higher for students in the lockdown cohort, who had access to online resources during the teaching phase and were also examined online: the pass rates for exams 2a (face-to-face) and 2b (online) were, respectively, 69.8% and 89%) (*p* = 0.001) (Figure 4a).

Turning now to the evaluation of practical skills (Figure 4b), no significant difference was found in student outcomes for exam 3 (cells and tissues), where students were required to examine static images either in the classroom (exam 3a: 92.5% pass rate) or via Moodle (exam 3b: 90.9% pass rate). However, for exams 4 and 5 (identifying organs from static images and using a microscope, respectively), students who underwent the online assessments had much higher pass rates than those who completed in-person exams and the effect was most marked for exam 5, where students were required to use a microscope: for static image exams 4a (face-to-face) and 4b (online), the rates were 86.3% and 94.5%, respectively (*p* = 0.058), while for microscope exams 5a (face-to-face; OM) and 5b (online; VM), rates were 87.6% and 100%, respectively (*p* < 0.001).

With regard to the overall scores achieved by students in the two cohorts, considerably higher pass rates were recorded for those who had experienced the lockdown-adapted course and online assessment (94.5%) compared to those who had completed the traditional course and face-to-face evaluation procedures (78.5%) (Figure 4c). One interesting finding was the significant differences revealed in the scaled qualitative scores between face-to-face and online evaluation (*p* < 0.001). Specifically, the percentage of students achieving the ratings of good and merit increased significantly in the online assessment: ratings of good rose from 34.6% in face-to-face exams to 75.5% in online exams, while those for merit rose from 1.9% to 6.4% (Figure 4d).

**Figure 5:**
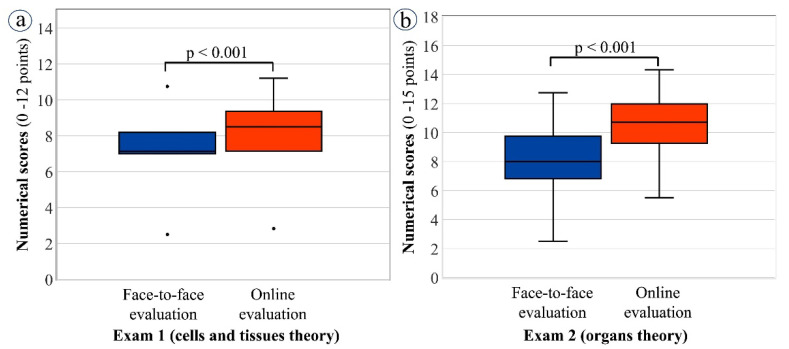
Box plots of median and interquartile range (IQR) for numerical scores achieved by students in theoretical exams (exams 1 and 2): face-to-face (2018–2019) and online (2019–2020) cohorts compared. (**a**) Exam 1 on cells and tissues: face-to-face (median = 7.13; IQR = 7–8.19) versus online (median = 8.51; IQR = 7.15–9.37) evaluation; (**b**) Exam 2 on organs: face-to-face (median = 8; IQR = 6.81–9.75) versus online (median = 10.72; IQR = 9.26–11.98) evaluation. Non-parametric Mann–Whitney U test was applied for statistical analysis.

It is worth noting that although the pass rate for exam 1 (cells and tissues theory) was similar for both the face-to-face and the online student cohorts, the median numerical scores achieved by students were significantly better for the online cohort compared to the students examined in-person (*p* < 0.001) (Figure 5).

**Figure 6:**
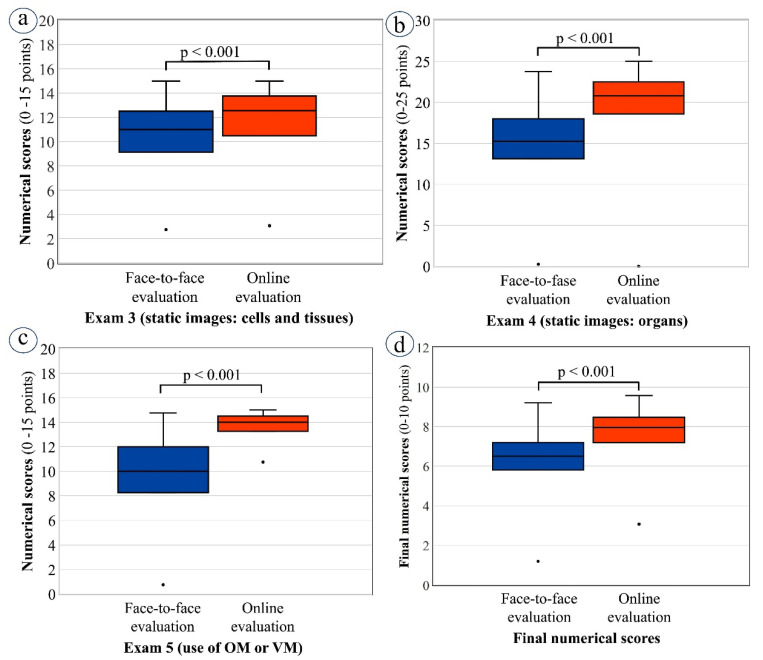
Box plots of median and interquartile range (IQR) of numerical scores achieved by students in theoretical exams (exams 1 and 2): face-to-face (2018–2019) and online (2019–2020) cohorts compared. (**a**) Exam 3 on the identification of cells and tissues with use of static images: face-to-face (median = 11.00; IQR = 9.13–12.50) versus online (median = 12.55; IQR = 10.49–13.75) evaluation; (**b**) Exam 4 on the identification of organs using static images: face-to-face (median = 15.25; IQR = 13.13–18.00) versus online (median = 20.80; IQR = 18.59–22.50) evaluation; (**c**) Exam 5: the identification and description of organs using either optical microscopy (median =10; IQR = 8.25–12.00) or virtual microscopy (median = 14.00; IQR = 13.25–14.50); (**d**) Final numerical scores in CH: face-to-face (median = 6.50; IQR = 5.80–7.20) versus online (median = 7.96; IQR = 7.19–8.47) evaluation. Non-parametric Mann–Whitney U test was applied for statistical analysis. OM, optical microscopy; VM, virtual microscopy.

Referring to Figure 6, a similar pattern of results was seen for several other exams: the practical exams based on examination of static images; exam 3 on cells and tissues (Figure 6a); exam 4 on organs (Figure 6b); exam 5, where the face-to-face cohort used OM while the online cohort used VM (Figure 6c); and for students’ final scores (Figure 6d). The threshold for statistical significance, *p* < 0.001, was reached in all cases.

### 3.3. Results of the Satisfaction Survey

Two satisfaction surveys were used: one to assess students’ perceptions of the combined use of OM and VM, which was given to both student cohorts (2018–2019 and 2019–2020), and a second to assess students’ opinions of the sole use of VM, which was only given to the 2019–2020 cohort. This second survey only applied to the 2019–2020 cohort and focused on their experiences during the lockdown-adapted part of the course, when organ micro-anatomy was taught. The results of both surveys are collated in Table 3. The satisfaction surveys revealed that students found the VM to be a very helpful resource, not just during the lockdown but also to complement conventional microscopy in face-to-face teaching (Table 3). However, more than 93% of students who experienced the face-to-face course and 72.4% of those who took the lockdown-adapted course believed that VM cannot completely replace the use of the OM in the practical teaching of CH (question 10, Table 3). Furthermore 100% of the lockdown cohort of students expressed the opinion that a combination of VM and OM was a good teaching method for learning histology (question 9, Table 3). Regarding the usefulness of VM for practical learning in CH during the lockdown, in answer to the item: “You have found the VM helpful for practical learning of organs”, most students answered, “strongly agree” (65.5%) or “agree” (31%) with 0% disagreeing (question 6, Table 3). However, in answer to the same item, a small percentage of this cohort felt that VM had not been useful in the teaching of cells and tissues prior to lockdown (10.3%) and a few of the 2018–2019 cohort felt that it had not been useful in their course (6.5%).

## 4. Discussion

In this study, we both assess and compare, for the first time, the theoretical and practical knowledge acquired by veterinary medicine students on cytology and histology, through a description of methodologies used prior to (traditional course) and during (lockdown-adapted course) the COVID-19 lockdown. The COVID-19 pandemic, and particularly the associated lockdown, gave rise to important changes in every sector, including education. At universities across the world, students and lecturers suddenly found themselves locked in their homes and unsure as to how they should go on with their academic activities. Teaching and learning had to continue, so lecturers were required to quickly adapt their traditional presential teaching methods and evaluation processes to the emerging remote methodology [5]. A lack of both experience and digital competence among many lecturers generated an atmosphere of uncertainty, and lecturers faced a huge responsibility in choosing the best didactic and assessment methods to ensure students’ successful academic achievement [10,11]. Furthermore, the changes in methodology that were required to adapt teaching and learning to the new pandemic situation also had significant psychological and academic impacts on students. This is perhaps because, although most students belong to a generation who consider themselves digital natives, their expertise is mostly focused on the social aspects of information and communication technology (ICT), and not all students have sufficient digital competence for academic purposes. Indeed, the definition of “digital competence” is the ability to make confident, critical, and creative use of ICT to achieve goals in a suite of areas including not only inclusion and/or participation in society but also work, employability, learning, and leisure [12]. In this regard, the adapted methodology was also useful for the students’ acquisition of transversal competencies such as the use of ICT to improve learning.

The results of this study, which were based on the experiences of veterinary medicine students on the CH course, suggest that student outcomes were not negatively affected by the lockdown-adapted methodology that was implemented to cope with the COVID-19 crisis. In fact, academic outcomes for the 2019–2020 student cohort were improved compared to those obtained by the previous cohort, who experienced the traditional, face-to-face course methodology. However, it is interesting to assess the impact on the teaching–learning that each of the various resources used during lockdown may have had, and their students’ ability to acquire the required competencies in CH. Online learning has come to stay, and will continue to be a major part of many university activities; thus, this assessment is necessary to identify potential weaknesses in new methodologies to improve online provisions in university teaching.

A particularly interesting finding in this study concerns students’ acquisition of theoretical knowledge. The pass rates for exams 1 and 2 (theoretical exams on cells and tissues, and organs, respectively) showed very different trends when comparing the face-to-face and online versions. The pass rates for the face-to-face and online versions of exam 1 were very similar (exam 1a (face-to-face): 80.6% versus exam 1b (online): 79.1%), while, for exam 2, the online exam pass rate (exam 2b: 89.0%) was significantly higher than that for its face-to-face version (exam 2a: 69.8%). One factor that may explain this difference is the teaching received by the two student cohorts for the examined material. Specifically, both student cohorts received presential teaching for the material examined in exams 1a and 1b, whereas the material examined in exam 2b (highest pass rate) was taught online. These results indicate that student outcomes are linked not only to assessment methods, but also to teaching methods [5,10].

Our results suggest that the good academic results we saw in 2019–2020 were partly due to the use of VM during the COVID-19 lockdown. During lockdown, the VM tool became the main resource, used not only for teaching and learning but also for student evaluation, since conventional OM was not available. In brief, VM involves the use of imaging software to examine the digitized optical microscope slides created using specialized slide scanners. VM software allows the user to navigate, change magnification, focus, and mark areas of interest on the slides. Several studies have shown that students obtained better learning outcomes when they were able to use VM rather than just OM [6,13,14,15], proving the usefulness of this technology for teaching and learning [3,4,8]. However, its helpfulness as part of student evaluation, at least in the format employed in this study (i.e., as part of online exams in Moodle), is not clear from our results. Of particular concern is that the significant difference in the pass rate for students taking the face-to-face and online formats of exam 5 (87.6% and 100%, respectively) might not reflect the real level of knowledge acquired by some students. This questionnaire involved embedded digital images and essay-style questions where students were required to write free-text descriptions of the organ structures displayed and, during the manual marking of this exam, lecturers noted that exactly the same descriptions and the same mistakes appeared in different exams. This suggests that students may have prepared their answers in advance and then copied and pasted them into the Moodle answer box. In this instance, a change from essay-style questions to a more controlled format could perhaps reduce this vulnerability. Despite this weakness, identified in the evaluation process, the usefulness of VM as a key tool to the acquisition of histology skills cannot be underemphasized; it was a well-used resource for students throughout lockdown and particularly during the exam period.

There are several elements that may have made online teaching more productive for the students in our study. In addition to the use of VM mentioned above, another set of resources was the pre-recorded lectures used as a substitute for classroom teaching. These were not used at all in the traditional, pre-COVID course (2018–2019), nor were they part of the teaching for cells and tissue theory during the pandemic course (2019–2020). A key advantage of the pre-recorded lectures was that they were available over a long period of time for students to watch whenever they wished, and this flexibility may have helped many students with time management. The online self-assessment tests included after each pre-recorded lecture, set, as explained, to stand in for class attendance, encouraged students to maintain a consistent workload.

The conclusion that face-to-face versus online methodologies lead to different academic outcomes for students is further supported by the overall course pass rate as well as the numerical and qualitative scores obtained by our two student cohorts. Overall course pass rate, and the numerical and qualitative scores, suggest that students achieved better academic results for the lockdown-adapted course, and this improvement could be attributed to the introduction of new teaching resources, particularly VM, pre-recorded lectures, and the online self-assessment tests provided for each topic.

However, it is worth stressing that it is important to consider the weakness detected in the online methodology for student evaluation. These problems can, of course, be extended to the many other exams conducted online during the lockdown and include, on the one hand, the possibility that some students will have accessed material to help solve exam questions and may have communicated among themselves, and on the other hand, the difficulty of ensuring the true identity of the person carrying out a given examination. Our results suggest that, in the CH course, lecturers’ efforts to avoid the problems outlined were not sufficient and, as a result, there are remaining issues with evaluation that require attention. Indeed, the ongoing use of online methods to assess students in CH is contingent on the development of more robust forms of assessment and methods for the identification of students. Among the strategies that might be introduced, we would suggest a greater variety of test types and, for student identification, the use of facial recognition tools (e-proctoring). This latter method is still under debate due to the current data protection legislation (Ley Orgánica 3/2018; Reglamento (UE) 2018/1725 del Parlamento Europeo y del Consejo, de 23 de octubre de 2018) [16].

We need to deepen our understanding of students’ perceptions of educational innovations such as those discussed in this work so that they can be improved using students’ suggestions and opinions [17]. In this study, we used a questionnaire to survey students’ opinions of VM and results revealed our students had a good perception of VM, as did the students surveyed in several other studies [4,14]. As in other institutions, our decision to replace conventional optical microscopy with VM was made out of necessity due to the lockdown requirements during the COVID-19 pandemic [8]. Students from both our cohorts (2018–2019 and 2019–2020) generally gave positive feedback about VM, with many expressing the view that VM was easy to use and a helpful tool in teaching practical skills for CH. Nevertheless, a high percentage of students in the 2019–2020 cohort believed that VM should not ever totally replace conventional microscopy. This is an interesting finding, as this group experienced both the use of VM as a complement to OM before the lockdown and moved to using only VM after the lockdown. The many advantages of using this emerging technology for educational purposes have been described in the previous literature [15], and they include remote access, an improved educational experience, and the guarantee of equal and consistent slide quality for all students. A particular advantage of VM is that this technology allows for unrestricted access to the material—slides are available for examination anytime, and from anywhere—which gives students the ability to better organize their study time [18]. It is also easy to maintain, provides students with clearer images, and, furthermore, enables collaborative learning (many students can view the same image at once), which may help students better retain theorical concepts of morphology and structure. In practice, however, students may feel uncomfortable if they are not given the opportunity to use conventional OM at some point in their course. In addition, at the beginning of their training, students will find some structures hard to identify [19]; therefore, it is important that lecturers are on hand to use their histology expertise to assist students in the interpretation of virtual images. It should also be noted that adjustment to new technologies takes time, and individual users will have different rates of adaptation—some slower than others—to new methodologies. VM can also present some disadvantages since it requires significant initial financial investment by the institution.

Finally, the role of lecturers in the learning process must not be underestimated. Lecturers play a key role in stimulating students’ interest and creating an environment in which students’ desire to learn is driven by a wish to acquire the knowledge and skills necessary for their professional future rather than just to pass the exam. This motivation is more easily fostered in a presential teaching environment than in online modalities and this is a point that will be addressed by the lecturers’ working groups. Unfortunately, our study was not designed to investigate how students perceived the role of their lecturers during the lockdown; nevertheless, the available channels of communication were well-used: message logs show continuous communication throughout lockdown, with a peak during the exam period.

## 5. Conclusions

In conclusion, the lockdown-adapted, online teaching-learning methodology used during the 2019–2020 pandemic had a positive effect on the academic outcomes for veterinary-CH students in this cohort. It is suggested that the use of explanatory pre-recorded lectures with theoretical and practical contents, continuous self-assessment of students through online tests, enabling students to monitor their work, and provision of VM as the key tool for practical training were of particular importance in students’ academic success.

However, weaknesses were identified in the online evaluation methods used in this study, and it was felt that they were not necessarily a good reflection of students’ true knowledge. Improvements to address the shortcomings of online evaluation include the use of identity verification tools (e-proctoring); conduction of different types of online tests, perhaps the use of questions requiring more reasoning and less reliance on recall, and oral exams; and, in certain situations, a final presential examination might be appropriate. To ensure the ongoing effectiveness of online teaching, it is necessary to enhance the university staff training program to place more emphasis on ICT skills and teaching methods that improve student engagement.

## Figures and Tables

**Table 1:** Theoretical and practical teaching methods in cytology and histology: A description and comparison of teaching methods used prior to (traditional course) and during COVID-19 lockdown (lockdown-adapted course).

Course Section	Teaching Methods on the Traditional Course: Face-to-Face (2018–2019)	Teaching Methods on the Lockdown Adapted Course: Face-to-Face and Online (2019–2020)
Cytology (theory)	8 × 1-h face-to-face classes in classroom (teaching contents in pdf files available on Moodle)	
Histology technique (theoretical and practical)	1 × 1-h face-to-face class in histology laboratory in groups of 10 students (theoretical contents in pdf files available in Moodle) 8 × 2-h face-to-face classes in microscopy laboratory	
Tissue histology (theoretical and practical)	Groups of 30–40 students Teaching contents in pdf files available in Moodle Histological slides available for examination of tissues using OM Complementary VM	
Organ microscopic anatomy (theoretical and practical)	- 14 × 2-h face-to-face classes - Groups of 30–40 students - Teaching contents in pdf files available in Moodle	- 14 online classes using pre-recorded lectures available in Moodle - No student groups - Teaching contents in pdf files available in Moodle
	1. Microscopy laboratory - Histological slides available for visualization of organs using OM 2. Moodle - Digital images for visualization of organs using VM (complementary tool)	1. Moodle - Digital images for visualization of organs using VM

VM, virtual microscopy. OM, optical microscopy.

**Table 2:** Evaluation of students’ acquired theoretical and practical knowledge on cytology and histology: A description of evaluation methods used prior to (traditional course) and during the COVID-19 lockdown (lockdown-adapted course). Images from (a) to (f) show student evaluation methods used in both courses.

Course Exam	Assessment Procedures for Traditional Course: Face-to-Face (2018–2019)	Assessment Procedures for Lockdown-Adapted Course: Face-to-Face and Online (2019–2020)
THEORY Exam 1: Cytology and histology (cells and tissues) Exam 2: Microscopic anatomy (organs)	Two presential written exams: Short answer (essay) ^a^, fill in the gaps ^a^, true false ^a^ and matching ^b^ questions. 1. Cytology and histology (exam 1a): 12 questions; 1 h 2. Microscopic anatomy (exam 2a): 15 questions; 1 h 30 min	Two online exams via Moodle: Short answer (essay) ^a^, fill in the gaps ^b^, true false ^a^, matching ^b^ and multiple-choice ^b^ questions. 1. Cytology and histology (exam 1b): 14 questions; 30 min 2. Microscopic anatomy (exam 2b): 15 questions; 35 min
PRACTICAL SKILLS Exam 3: Cytology and histology (cells and tissues)	One presential written exam: 1. Identification of cells and tissues (exam 3a): 15 projected static images; short answer (essay) ^a^ questions; 18-min time limit. 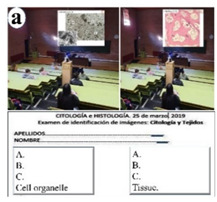	One online exam via Moodle: 1. Identification of cells and tissues (exam 3b): 15 static images; short answer (essay) ^a^ and matching ^b^ questions; 18-min time limit. 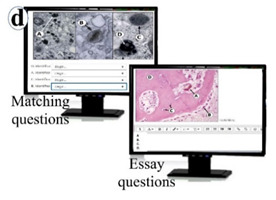
PRACTICAL SKILLS Exams 4 and 5: Microscopic anatomy (organs)	Two presential written exams: 1. Identification of organs (exam 4a): 25 projected static images; short answer (essay) ^a^ questions; 30-min time limit. 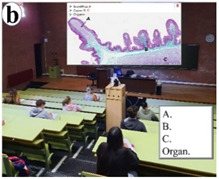 2. Identification and description of organs (exam 5a): 6 slides of organs examined using optical microscope; essay questions ^a^; 55-min time limit. 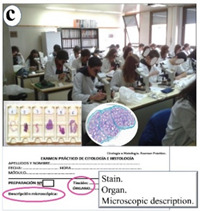	Two online exams via Moodle: 1. Identification of organs (exam 4b): 25 static images; short answer (essay) ^a^ and matching ^b^ questions; 30-min time limit. 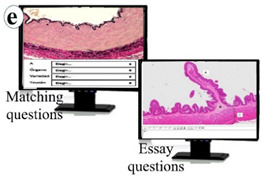 2. Identification and description of organs (exam 5b): 5 virtual slides of organs examined using VM; essay questions ^a^; 45-min time limit. 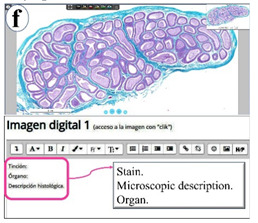

^a^ Answer options are not shown to students. ^b^ Answer options are shown to students.

**Table 3:** Student satisfaction survey: cohorts from 2018–2019 and 2019–2020 were asked to rate their opinions of the combined use of optical and virtual microscopy (VM) in CH. For the 2019–2020 cohort this focused on their pre-COVID experiences of the face-to-face teaching of cells and tissues. The 2019–2020 cohort was asked additionally to rate their opinions of VM used alone, as was the case in the lockdown-adapted section of their course, when organ micro-anatomy was taught.

Items and Values	2018–2019 Course (*n* = 31)	2019–2020 Course (*n* = 29)	
Sex			
Female	93.5% (29/31)	82.8% (24/29)	-
Male	6.5% (2/31)	17.2% (5/29)	-
2. You had some knowledge of animal histology before taking this course.			
Strongly agree	0	0	-
Agree	0	0	-
Neutral	16.1% (5/31)	13.79% (4/29)	-
Disagree	83.9% (26/31)	86.2% (25/29)	-
Items and values	Opinions based on experience of the traditional CH course: face-to-face teaching of cells, tissues, and organs	Opinions based on pre-COVID experience: face-to-face teaching of cells and tissues	Opinions based on lockdown experience: online teaching of organs
3. You have used the VM prior to the classes for their preparation.			
Yes	12.9% (4/31)	17.24% (5/29)	51.7% (15/29)
No	87.1% (27/31)	82.8% (24/29)	48.3% (14/29)
4. You have used the VM to prepare the online questionnaires and the exams.			
Yes	87.1% (27/31)	55.2% (16/29)	89.7% (26/29)
No	12.9% (4/31)	44.8% (13/29)	10.3% (3/29)
5. VM software is easy to use.			
Yes	83.9% (26/31)	100% (29/29)	-
No	16.1% (5/31)	0	-
6. You have found the VM helpful for practical learning in CH.			
Strongly agree	71.0% (22/31)	38% (11/29)	65.5% (19/29)
Agree	12.9% (4/31)	31% (9/29)	31.0% (9/29)
Neutral	9.7% (3/31)	20.7% (6/29)	3.5% (1/29)
Disagree	6.5% (2/31)	10.3% (3/29)	0
7. You have found the VM helpful in theoretical learning of CH.			
Strongly agree	38.7% (12/31)	-	
Agree	29.0% (9/31)	-	
Neutral	22.6% (7/31)	-	
Disagree	9.7% (3/31)	-	
8. Identification of cells, tissues, and organs with VM is easy.			
Yes	-	-	93.1% (27/29)
No	-	-	6.9% (2/29)
9. Combination of VM and the use of light microscopy is a good teaching method for learning histology.			
Yes	-	-	100% (29/29)
No	-	-	0
10. VM can completely replace the use of the optical microscope in the teaching of CH.			
Yes	6.5% (2/31)	-	27.6% (8/29)
No	93.5% (29/31)	-	72.4% (21/29)
11. Free comments	No	During the face-to-face teaching, I have not used VM, and I studied only by notes (badly done because it is very enlightening).	Thanks to the lecturers for having accompanied us tirelessly in this very difficult time of lockdown

Results were expressed as a percentage of students (number/total number of students responding to the survey). VP, virtual microcopy. CH, Cytology and Histology.

## Data Availability

Not applicable.
